# Hand Book of Materia Medica, Pharmacy and Therapeutics

**Published:** 1891-12

**Authors:** 


### REVIEWS.

Hand Book of Materia Medica, Pharmacy and Therapeutics, including the Physiological Action of Drugs, the Special Therapeutics of Disease, Official and Practical Pharmacy and Minute Directions for Prescription Writing. By Sam'l O. L. Potter, A.M., M.D. (Jeff'n), M.R.C.P. (Lond.), Professor of Theory and Practice of Medicine in the Cooper Medical College of San Francisco. *Third Edition Revised.* Philadelphia: P. Blakiston, Son & Co., 1012 Walnut Street, 1891.

The modern student of medicine, human or veterinary, cannot complain of lack of facilites for the prosecution of his special work, since every year a new treatise on questions pertaining to the subject matter of his studies is issued from the press, each exhibiting some special feature of recommendation, or some salient point of interest. And this is as it should be, for the ever accumulating mass of materials which modern research has brought to light needs a constant systematization in order that the student may not be lost in a wilderness of fragmentary facts. Of recent works that have appeared with this important object in view, none will recommend itself to the student or practitioner more strongly than the excellent treatise of Dr. Potter. It is fully up to the most advanced line of current medical thought and bristles with interesting facts and observations. But the chief features of its excellence lies in its faultless arrangement. The initial chapters on the administration and classification of medicines are brief in statement, pithy in matter, and lucid in arrangement. Here the student becomes acquainted, almost at a glance with the methods by which the animal economy falls under the influence of drugs, and the chief agents that produce effects on the various organs of the body. The action of each leading pharmaceutical preparation is briefly stated in larger type and special remarks follow in smaller print, so that the student can readily discriminate between what appeals to his attention with reference to its importance and what he is not so peremptorily obliged to remember. This same convenience of arrangement pervades the whole book and invites the attention of the students by its attractiveness. The latest additions to the armentarim of the physician are here given and the most interesting details are added. The one page devoted to the consideration of antidotes and antagonists is replete with valuable information and will supply more knowledge to the student than many pages of recognized standard works. Perhaps one of the most difficult points with which the young student of medicine has to struggle is the art of writing prescriptions, of determining the doses, of fixing the proportions and ascertaining the separate and combined effects of various drugs.

Many text-books, with a view to overcoming this difficulty, append to their pages a list of prescriptions made out in full with directions accompanying and a statement of the circumstances under which they may be profitably employed. This proceeding savors of impiricism leads to routinism and is eminenly unscientific. Dr. Potter deals with the difficulty in a far more logical and, at the same time, far easier manner.

He begins his chapter on pharmacy and prescription writing with clear and concise definitions of all the terms that constantly confront the student; he then briefly discusses the natural history and chemical constitution of each substance, points out its physiological and pathological properties, assigns the dose and considers its synergic and antagonistic qualities. The author deplores the fact that so many enter upon the study of medicine without a sufficient preparatory knowledge of the humanities, and he wisely recommends those who possess no knowledge or an imperfect knowledge of Latin, to write their prescriptions in English. It is better to avow ignorance than to put on the caps and bells of a Cagliostro. We take great pleasure in cordially recommending Dr. Potter's excellent treatise to the medical profession and above all to students of human and veterinary medicine. C. M. O'L.

